# *WISP1* Polymorphisms Contribute to Platinum-Based Chemotherapy Toxicity in Lung Cancer Patients

**DOI:** 10.3390/ijms151121011

**Published:** 2014-11-14

**Authors:** Juan Chen, Jiye Yin, Xiangping Li, Ying Wang, Yi Zheng, Chenyue Qian, Ling Xiao, Ting Zou, Zhan Wang, Junyan Liu, Wei Zhang, Honghao Zhou, Zhaoqian Liu

**Affiliations:** 1 Department of Clinical Pharmacology, Xiangya Hospital, Central South University, Changsha 410008, China; E-Mails: cj1028@csu.edu.cn (J.C.); yinjiye2005@sina.com (J.Y.); xylxping@126.com (X.L.); lizhi1990312@163.com (Y.Z.); qianchenyue0824@foxmail.com (C.Q.); xiaolingcsu@csu.edu.cn (L.X.); blingbreeze@163.com (T.Z.); yjsd2003@163.com (W.Z.); HHzhou2003@163.com (H.Z.); 2 Institute of Clinical Pharmacology, Central South University, Hunan Key Laboratory of Pharmacogenetics, Changsha 410078, China; 3 The Affiliated Cancer Hospital of XiangYa School of Medicine, Central South University, Changsha 410014, China; E-Mail: wy552@hotmail.com; 4 Department of Oncology, Xiangya Hospital, Central South University, Changsha 410008, China; E-Mail: wan0916wan@163.com; 5 Xiangya School of Medicine, Central South University, Changsha 410008, China; E-Mail: liujunyana@126.com

**Keywords:** *WISP1*, lung cancer, genetic polymorphism, chemotherapy toxicity

## Abstract

Platinum-based chemotherapy toxicity is always one of the serious problems from which lung cancer patients suffer. The genetic polymorphism of *WISP1* was revealed to be associated with susceptibility and platinum-based chemotherapy response in our previous studies. In this study, we aimed to investigate the relationship of *WISP1* genetic polymorphisms with platinum-based chemotherapy toxicity in lung cancer patients. A total of 412 lung cancer patients were enrolled in this study, and 28 polymorphisms of the *WISP1* gene were genotyped by SequenomMassARRAY. We found that *WISP1* polymorphisms (rs2929965, rs2929969, rs2929970, rs2929973 and rs754958) were related to the overall chemotherapy toxicity of lung cancer in subgroup analyses. Rs16904853, rs2929970, rs2977549 and rs2977551 (*p* = 0.021, 0.028, 0.024, 0.048, respectively) polymorphisms were significantly associated with hematologic toxicity. Rs2929946, rs2929970, rs2977519, rs2977536, rs3739262 and rs754958 (*p* = 0.031, 0.046, 0.029, 0.016, 0.042, 0.035, respectively) polymorphisms were significantly associated with the gastrointestinal toxicity of lung cancer. Genotypes of *WISP1* may be novel and useful biomarkers for predicting platinum-based chemotherapy toxicity in lung cancer patients.

## 1. Introduction

In recent years, lung cancer has become the highest morbidity and mortality cancer among cancers in the world [1]. It consists of non-small cell lung cancer (NSCLC) and small cell lung cancer (SCLC). Most lung cancer patients were diagnosed at advance stages. They were not suitable for surgery, and chemotherapy was the best choice for them. Platinum-based chemotherapy is widely used for different cancers, especially cisplatinum, for the treatment of lung cancer [2]. However, chemotherapy resistance and toxicity are still serious problems from which lung cancer patients suffer. It was reported that chemotherapy efficacy and toxicity were related to many genes in multiple pathways, such as DNA repair, apoptosis, transportation, as well as the Wnt pathway [3,4,5,6,7]. Wnt-1 inducible signaling protein 1 (*WISP1*) is a target gene of the canonical Wnt signaling pathway [8]. Its expression was revealed to be associated with pulmonary fibrosis, ventilator-induced lung injury and lung cancer [9,10].

*WISP1* belongs to the CCN protein family, which consists of cysteine-rich 61 (*CYR61/CCN1*), connective tissue growth factor (*CTGF/CCN2*), nephroblastoma overexpressed (*NOV/CCN3*), *WISP1*, *WISP2* and *WISP3* [11,12,13]. It maps to chromosome 8q24.1–8q24.3 and has five exons and four introns [14]. It is a secreted matricellular protein and has four modules, namely insulin growth factor binding protein (IGFBP), Van Willebrand factor C (VWC), thrombospondin type I repeat domain (TSP) and C-terminal domain (CT) [11]. It is involved in diverse biological effects, such as cell proliferation, differentiation and survival, and is related to multiple pathologic processes, especially in lung diseases [9,15,16,17].

Nowadays, single-nucleotide polymorphisms (SNPs) play vital roles in the occurrence and development of diseases. Numerous recent studies have revealed that polymorphic genetic mutations contributed to the toxicity and chemotherapy resistance of lung cancer. Moreover, the polymorphisms of *WISP1* were discovered to be associated with diverse lung diseases [10,18]. In our previous study, *WISP1* polymorphisms were revealed to be related to susceptibility and the platinum-based chemotherapy response of lung cancer in Chinese patients [19].

In order to further investigate the role of *WISP1* polymorphisms in the chemotherapy toxicity of lung cancer, we selected 28 SNPs of the *WISP1* gene and genotyped in lung cancer patients to explore the association between *WISP1* polymorphisms and platinum-based chemotherapy toxicity.

## 2. Results

### 2.1. Clinical Characteristics and Toxicity Outcomes of Subjects and Genotyping

A total of 412 lung cancer patients were enrolled in our study. Their clinical characteristics are summarized in Table 1. There were 288 NSCLC patients and 124 SCLC patients. All 412 patients received at least two cycles of platinum-based chemotherapy, and severe toxicity occurred in 163 (39.6%) of them. Seventy one (22.1%) patients suffered hematologic toxicity; 72 (22.3%) patients suffered gastrointestinal toxicity; and 20 (4.8%) patients suffered both hematologic and gastrointestinal toxicity. All 28 SNPs of the *WISP1* gene were genotyped by Sequenom’s MassARRAY system. As shown in Table 1, the call rate of the SNPs ranged from 95.63% to 99.76%, and the MAF (minor allele frequency) of each SNP was more than 5%. The genotype distribution of 28 SNPs in the severe toxicity group and non-severe toxicity group were in accordance with the Hardy-Weinberg equilibrium.

**Table 1:** The polymorphisms of WISP1 examined in this study.

Polymorphisms	Alleles	Call Rate (%)	MAF ^†^
rs10956696	C/T	98.79	0.10
rs10956697	A/C	96.60	0.33
rs11778573	G/T	98.30	0.41
rs16893344	C/T	98.30	0.13
rs16904853	C/T	95.63	0.47
rs2013146	C/T	98.54	0.35
rs2929946	A/G	97.82	0.08
rs2929965	C/T	97.57	0.42
rs2929969	A/G	97.57	0.32
rs2929970	A/G	99.76	0.37
rs2929973	G/T	97.33	0.34
rs2929986	C/T	97.57	0.37
rs2977519	A/T	95.87	0.31
rs2977529	A/T	99.27	0.19
rs2977530	A/G	98.54	0.45
rs2977536	C/G	98.30	0.33
rs2977537	A/G	98.30	0.47
rs2977549	C/T	98.79	0.38
rs2977551	T/C	98.79	0.38
rs3739262	C/T	97.33	0.15
rs4330674	T/C	99.76	0.15
rs62514003	T/C	98.79	0.14
rs62514004	G/A	96.36	0.12
rs72731505	C/T	98.06	0.38
rs72731507	A/G	99.76	0.09
rs754958	C/T	98.79	0.32
rs7828685	G/A	98.78	0.29
rs7843546	C/T	99.75	0.49

**^†^** Minor allele frequency.

### 2.2. Association between WISP1 Polymorphisms and the Severe Toxicity of Platinum-Based Chemotherapy in Lung Cancer Patients

**Figure 1:**
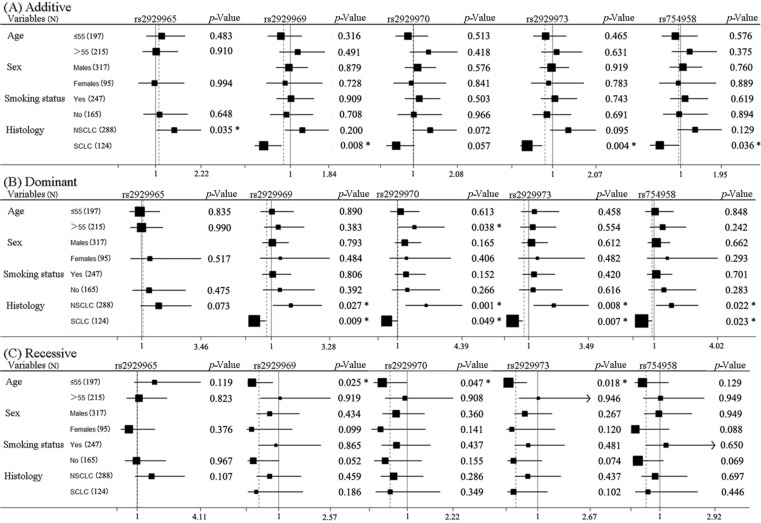
Stratification analyses of the associations of *WISP1* rs2929965, rs2929969, rs2929970, rs2929973 and rs754958 polymorphisms with the overall chemotherapy toxicity of lung cancer. Each box and horizontal line represents the values of OR and 95% CI. NSCLC, non-small cell lung carcinoma; SCLC, small cell lung carcinoma. * *p* < 0.05.

The 28 polymorphisms of *WISP1* were not statistically significantly related to increased risk of overall severe toxicity in all three models (Table S1). However, there were five SNPs related to the toxicity in subgroup analyses. As shown in Figure 1, *WISP1* rs2929965 polymorphism was related to the overall toxicity of NSCLC patients in the additive model; *WISP1* rs2929969 and rs2929973 were related to the overall toxicity of SCLC in additive and dominant models and related to the toxicity of NSCLC in the dominant model. Additionally, they were also related to overall toxicity in lung cancer patients ≤55 years old in the recessive model. *WISP1* rs2929970 was related to overall toxicity in patients >55 years old and in NSCLC and SCLC patients in the dominant model, as well as in patients ≤55 years old in the recessive model. *WISP1* rs754958 was related to the overall toxicity of SCLC patients in additive and dominant models and related to the overall toxicity of NSCLC in the dominant model. In conclusion, lung cancer patients ≤55 years old carrying the A allele of (rs2929969, rs2929970) or the G allele of rs2929973; patients >55 years old carrying the G allele of rs2929970; NSCLC patients carrying the T allele of (rs2929965, rs2929973, rs754958) or the G allele of (rs2929969, rs2729970); SCLC patients carrying the A allele of (rs2929965, rs2929970), or the G allele of rs2929973, or the C allele of rs754958 suffered more risk of Grade 3 or 4 toxicity overall for platinum-based chemotherapy.

### 2.3. Association between WISP1 Polymorphisms and the Hematologic Toxicity of Platinum-Based Chemotherapy in Lung Cancer Patients

**Figure 2:**
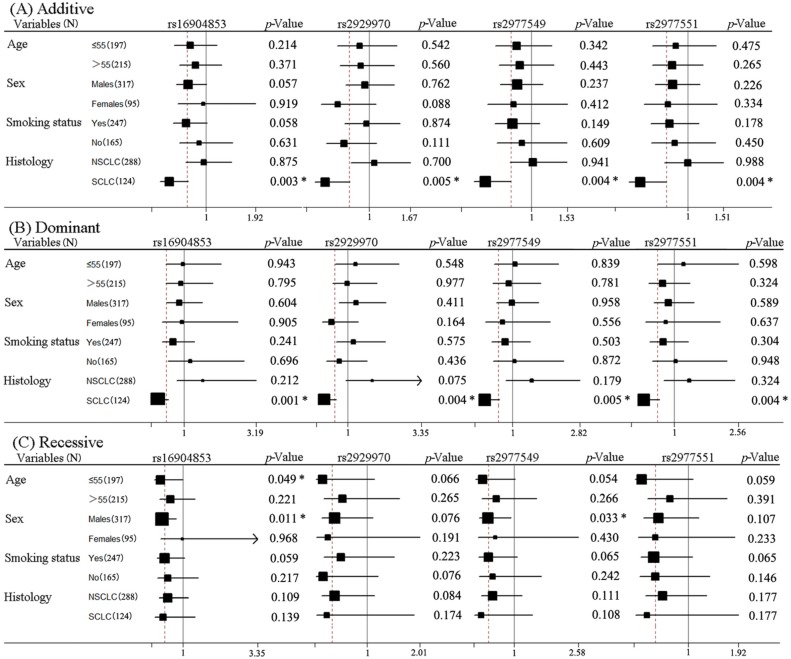
Stratification analyses of the associations of *WISP1* rs16904853, rs2929970, rs2977549 and rs2977551 polymorphisms with the hematologic toxicity of lung cancer. Each box and horizontal line represents the values of OR and 95% CI. NSCLC, non-small cell lung carcinoma; SCLC, small cell lung carcinoma. * *p* < 0.05.

As shown in Table 2, *WISP1* polymorphisms (rs16904853, rs2929970, rs2977549, rs2977551) were significantly associated with the hematologic toxicity of platinum-based chemotherapy of lung cancer patients in the recessive model. The results of subgroup analyses of the four SNPs are demonstrated in Figure 2. *WISP1* rs16904853 and rs2977549 were associated with the hematologic toxicity of SCLC in additive and dominant models and associated with the toxicity of patients ≤55 years old in the recessive model. *WISP1* rs16904853 was also associated with the hematologic toxicity of the lung cancer in males in the recessive model. *WISP1* rs2929970 and rs2977551 were associated with the hematologic toxicity of SCLC in additive and dominant models. All of the results showed that individuals carrying the C allele of (rs16904853, rs2977549), or the A allele of rs2929970, or the T allele of rs2977551 had an increased risk of hematologic toxicity for platinum-based chemotherapy.

**Table 2:** Association of rs16904853, rs2929970, rs2977549 and rs2977551 with hematologic toxicity in lung cancer patients.

SNPs	Non-3 or 4 Grade Toxicity (11/12/22 ^†^)	3 or 4 Grade Toxicity (11/12/22)	MAF		Additive		Dominant		Recessive	
			Non-3 or 4 Grade Toxicity	3 or 4 Grade Toxicity	OR (95% CI)	*p*	OR (95% CI)	*p*	OR (95% CI)	*p*
rs16904853	83/152/75	26/51/11	0.49	0.41	0.74 (0.52–1.05)	0.087	0.87 (0.52–1.47)	0.607	0.45 (0.23–0.89)	0.021 *
rs2929970	123/145/47	34/49/5	0.38	0.34	0.82 (0.57–1.17)	0.276	1.02 (0.63–1.65)	0.944	0.34 (0.13–0.89)	0.028 *
rs2977549	58/136/124	7/45/36	0.40	0.34	0.78 (0.55–1.10)	0.153	0.92 (0.57–1.49)	0.745	0.39 (0.17–0.88)	0.024 *
rs2977551	122/142/52	38/44/7	0.39	0.33	0.76 (0.54–1.08)	0.128	0.84 (0.52–1.36)	0.486	0.43 (0.19–0.99)	0.048 *

^†^ Wild-type/heterozygote/homozygote; * *p* < 0.05.

### 2.4. Association between WISP1 Polymorphisms and the Gastrointestinal Toxicity of Platinum-Based Chemotherapy in Lung Cancer Patients

**Figure 3:**
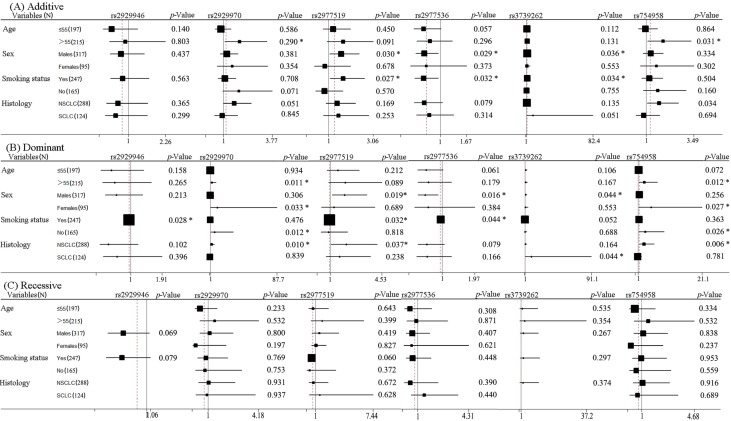
Stratification analyses of the associations of *WISP1* rs2929946, rs2929970, rs2977519, rs2977536, rs3739262 and rs754958 polymorphisms with the gastrointestinal toxicity of lung cancer. Each box and horizontal line represents the values of OR and 95% CI. NSCLC, non-small cell lung carcinoma; SCLC, small cell lung carcinoma. * *p* < 0.05.

As shown in Table 3, *WISP1* polymorphisms (rs2929946, rs2929970, rs2977519, rs2977536, rs754958) were significantly associated with gastrointestinal toxicity in lung cancer patients in the dominant model. *WISP1* rs2977536 and rs3739262 were significantly associated with gastrointestinal toxicity in the additive model. The results of subgroup analyses of the six SNPs are demonstrated in Figure 3. *WISP1* rs2929946 was associated with the gastrointestinal toxicity of smoking patients in the dominant model. *WISP1* rs2929970 was associated with the gastrointestinal toxicity of patients >55 years old in additive and dominant models, and it was related to the toxicity of female patients, nonsmoking patients and NSCLC patients in the dominant model. *WISP1* rs2977519 and rs2977536 were associated with the gastrointestinal toxicity of male patients and smoking patients in additive and dominant models, and rs29277519 was also associated with the toxicity of NSCLC in the dominant model. *WISP1* rs3739262 was associated with the gastrointestinal toxicity of male patients in additive and dominant models, and it was also related to the toxicity of smoking patients in the additive model and of SCLC in the dominant model. *WISP1* rs754958 was associated with the gastrointestinal toxicity of patients >55 years old and NSCLC in additive and dominant models, and it was also associated with the toxicity of female patients and nonsmoking patients in the dominant model. The subgroup analyses indicating that individuals with the A allele of (rs2929946, rs2977549), or the G allele of rs2929970, or the C allele of rs2977536, or the T allele of (rs2977549, rs3739262, rs754958) presented a greater risk of gastrointestinal toxicity for platinum-based chemotherapy.

**Table 3:** Association of rs2929946, rs2929970, rs2977519, rs2977536, rs3739262 and rs754958 with gastrointestinal toxicity in lung cancer patients.

SNPs	Non-3 or 4 Grade Toxicity (11/12/22 ^†^)	3 or 4 Grade Toxicity (11/12/22)	MAF		Additive		Dominant		Recessive	
			Non-3 or 4 Grade Toxicity	3 or 4 Grade Toxicity	OR (95% CI)	*p*	OR (95% CI)	*p*	OR (95% CI)	*p*
rs2929946	42/59/254	2/7/82	0.48	0.06	0.55(0.28–1.10)	0.093	0.42(0.19–0.92)	0.031 *	5.41(0.43–67.28)	0.190
rs2929970	130/140/42	27/54/10	0.20	0.41	1.27(0.88–1.84)	0.201	1.72(1.01–2.93)	0.046 *	0.87(0.40–1.86)	0.715
rs2977519	158/130/31	36/48/8	0.15	0.35	1.39(0.95–2.02)	0.089	1.75(1.06–2.91)	0.029 *	1.01(0.43–2.40)	0.980
rs2977536	125/152/32	48/30/8	0.16	0.27	0.64(0.43–0.96)	0.030 *	0.53(0.32–0.89)	0.016 *	0.70(0.29–1.66)	0.417
rs3739262	232/77/6	59/28/3	0.03	0.19	1.64(1.02–2.63)	0.042 *	1.70(0.99–2.91)	0.055	2.34(0.52–10.50)	0.266
rs754958	34/120/153	8/49/33	0.36	0.36	1.32(0.91–1.91)	0.150	1.74(1.04–2.91)	0.035 *	0.86(0.37–2.02)	0.732

**^†^** Wild-type/heterozygote/homozygote; * *p* *<* 0.05.

## 3. Discussion

In the current study, we examined the relationship of *WISP1* polymorphisms and platinum-based chemotherapy toxicity in Chinese lung cancer patients. Our results showed that *WISP1* SNPs (rs2929965, rs2929969, rs2929970, rs2929973, rs754958) contributed to the overall toxicity in different subgroups; *WISP1* SNPs (rs16904853, rs2929970, rs2977549, rs2977551) were related to hematologic toxicity, and *WISP1* SNPs (rs2929946, rs2929970, rs2977519, rs2977536, rs3739262, rs754958) were related to the gastrointestinal toxicity of platinum-based chemotherapy in lung cancer patients.

The Wnt signaling pathway regulates the transcription of many genes that are involved in cell proliferation, differentiation and the prevalence of cancers [20,21,22]. The canonical Wnt-β-catenin pathway is one of the three major Wnt signaling pathways, and it is involved in diverse cancers, such as hepatocellular carcinoma and esophageal squamous carcinoma [23,24]. *WISP1* is a downstream gene of the canonical Wnt-β-catenin pathway, and its mutations were reported to be associated with multiple diseases, including asthma, hypertension and spinal osteoarthritis [18,25,26]. Our previous studies revealed that *WISP1* genetic polymorphisms were related to susceptibility and the platinum-based chemotherapy response of lung cancer, and we hypothesized that WISP1 polymorphisms may also be associated with the chemotherapy toxicity of lung cancer [19]. In this study, we found 12 *WISP1* polymorphisms contributing to platinum-based chemotherapy toxicity. Five SNPs (rs2929969, rs2929970, rs2929973, rs2977549, rs2977551) were located in the 3’UTR of *WISP1*. rs2929970 and rs2929973 have been reported to be related to many diseases [18,25,26]. Polymorphisms in the 3’UTR of genes would be able to regulate gene expression. Four SNPs (rs2799519, rs3739262, rs2977536, rs2929946) were in the intron 1 of the *WISP1* gene. Mutations at exon-intron junctions, the branch point sequence and the polypyrimidine tract would disrupt the highly conserved and receptor sites. Especially, the first intron is significantly related to gene expression. Three SNPs (rs16904853, rs2929965, rs754958) were located in other introns; these polymorphisms may play important roles in the splicing process or the structure of proteins [27,28,29,30,31]. However, the detailed mechanisms about the functions of *WISP1* polymorphisms need further investigations.

Lung cancer patients ≤55 years old carrying the A allele of (rs2929969, rs2929970) or the G allele of rs2929973, patients >55 years old carrying the G allele of *WISP1* rs2929970, NSCLC patients carrying the T allele of (rs2929965, rs2929973, rs754958) or the G allele of (rs2929969, rs2929970), SCLC patients carrying the A allele of (rs2929969, rs2929970), or G allele of rs2929973, or the C allele of rs754958 presented more risk of overall severe toxicity of platinum-based chemotherapy. Lung cancer patients ≤55 years old carrying the C allele of rs16904853, male patients carrying the C allele of (rs16904853, rs2977549), SCLC patients carrying the C allele of (rs16904853, rs2977549), or the A allele of rs2929970, or the T allele of rs2977551 had more risk of hematologic toxicity of platinum-based thermotherapy. Lung cancer patients >55 years old carrying the G allele of rs2929970 or the T allele of rs754958, male patients carrying the T allele of (rs2977519, rs3739262) or the C allele of rs2977536, female patients carrying the G allele of rs2929970 or the T allele of rs754958, smoking patients carrying the A allele of rs2929946, or the T allele of (rs2977519, rs3739262), or the C allele of rs2977536, nonsmoking patients carrying the G allele of rs2929970 or the T allele of rs754958, NSCLC patients carrying the G allele of *WISP1* rs2929970 or the T allele of (rs2977519, rs754958) exhibited more risk of gastrointestinal toxicity of platinum-based chemotherapy.

All of these SNPs were discovered for the first time to be associated with the chemotherapy toxicity of lung cancer. In our previous studies, the genetic polymorphisms of *WISP1*, eukaryotic translation initiation factor 3 (*eIF3a*) and copper transport protein 1 (*CTR1*) were shown to be associated with platinum-based chemotherapy response or toxicity in lung cancer patients [32,33,34,35]. Most previous studies about the relationships of WISP1 polymorphisms and diseases were focused on the polymorphisms of *WISP1* rs2929973 and rs2929970. These two polymorphisms were located in the 3'UTR of the *WISP1* gene. Sunita *et al.* indicated that *WISP1* rs2929973 was associated with asthma and individuals carrying the G allele of rs2929973 conferring lower forced expiratory volume in the first second [18]. Yamada Y *et al.* reported that *WISP1* rs2929970 was associated with hypertension in men, and the men carrying the G allele of rs2929970 had higher blood pressure [25]. Moreover, Urano *et al.* demonstrated that *WISP1* rs2929970 was associated with spinal osteoarthritis in postmenopausal Japanese women, and the women carrying AA genotypes had significantly higher endplate sclerosis [26]. In our study, *WISP1* rs2929973 was related to the chemotherapy toxicity of SCLC; *WISP1* rs2929970 was associated with the overall toxicity, hematologic toxicity and gastrointestinal toxicity of lung cancer. Perhaps, the two SNPs play very important roles in the expression or function of the *WISP1* gene, and they are associated with multiple diseases.

However, there were several limitations to our study. Considering multiple-testing correction, which calculated the *p*-value of the SNPs by FDR-BH (Benjamini and Hochberg (1995) step-up False Discovery Rates control) correction, and that no SNPs remained significant, we consider that perhaps the sample size for the study was not large enough. The functional relevance of the identified polymorphisms that are associated with chemotherapy toxicity was not determined in our study. Finally, the validation of our results requires replication studies with other independent subjects.

In conclusion, our results showed that WISP1 polymorphisms of (rs16893344, rs2929970, rs2977549, rs2977551, rs2929946, rs2977519, rs2977536, rs3739262, rs754958, rs2929965, rs2929969, rs2929973) were significantly associated with the chemotherapy toxicity of lung cancer patients. Thus, we thought that the genotypes of *WISP1* may be used to predict the platinum-based chemotherapy toxicity in lung cancer patients.

## 4. Materials and Methods

### 4.1. Study Population and Treatments

We enrolled 412 lung cancer patients in this study. All individuals were provided written informed consent in compliance with the code of ethics of the World Medical Association (Declaration of Helsinki) before this study was initiated. Eligible subjects were from The Affiliated Cancer Hospital or Xiangya Hospital of Central South University (Changsha, Hunan, China) between November, 2011, and May, 2013. The patients that were eligible for the study had to meet the following criteria: (1) histologic or cytologic confirmed of lung cancer; (2) no prior chemotherapy; (3) Eastern Cooperative Oncology Group 0–2; (4) all patients were treated with platinum-based chemotherapy for at least two periods; and (5) organ function before chemotherapeutic treatment: liver function test (aspartate transaminase ≤1.5 × normal upper limit, alanine transaminase ≤1.5 × normal upper limit); blood test (leukocyte count ≥1.5 × 10^9^/L, neutrophil count ≥1.5 × 10^9^/L, platelet count ≥100 × 10^9^/L); kidney test (serum creatinine ≤1.5 × normal upper limit, creatinine clearance ≥60 mL/minute). Exclusion criteria included: (1) pregnancy or lactation; (2) active infection; (3) symptomatic brain or leptomeningeal metastases; and (4) previous or other concomitant malignancies. The study protocol was approved by the Ethics Committee of Xiangya School of Medicine, Central South University, with Registration Number CTXY-110008-1. We applied for this study for clinical admission in the Chinese Clinical Trial Register (Registration Number: ChiCTR-RNC-12002892). The chemotherapy regimens are listed in Table 4.

**Table 4:** Clinical characteristic of lung cancer patients.

Patients Characteristics	N (%)
Total No. of patients	412
Lung cancer	
NSCLC	288 (69.9)
SCLC	124 (30.1)
Age	
≤55	197 (47.8)
>55	215 (52.2)
Sex	
Male	317 (76.9)
Female	95 (23.1)
Smoking status	
Non-smoker	165 (40.0)
Smoker	247 (60.0)
ECOG PS ^†^ = 0–2	412 (100)
Stage	
I–II	20 (4.8)
III–IV	264 (64.1)
LD ^††^	56 (13.6)
ED ^†††^	65 (15.8)
Histology	
Adenocarcinoma	145 (35.2)
Squamous cell	143 (34.7)
Small cell	124 (30.1)
Chemotherapy regimens	
Platinum/gemcitabine	228 (55.3)
Platinum/paclitaxel	55 (13.3)
Platinum/navelbine	8 (1.9)
Platinum/etoposide	113 (27.4)
Platinum/irinotecan	8 (1.9)
Severe toxicity	
Total no.	163 (39.6)
Hematologic toxicity	91 (22.1)
Gastrointestinal toxicity	92 (22.3)

**^†^** Eastern Cooperative Oncology Group, performance status; **^††^** Limited Disease; **^†††^** Extensive Disease.

### 4.2. Data Collection

Clinical data of all patients were collected, including age, sex, smoking status, tumor histology, clinical stage and ECOG PS (Eastern Cooperative Oncology Group, Performance Status). According to National Cancer Institute Common Toxicity Criteria Version 3.0, the incidence of Grade 3 or 4 toxicity during first-line chemotherapy was collected. Toxicities that were associated with treatment that we collected included hematologic toxicity (anemia, leukopenia, neutropenia and thrombocytopenia) and gastrointestinal toxicity (nausea, vomiting and diarrhea). The investigators were blinded to the polymorphism status of the patients. Severe toxicity was defined as any Grade 3 or 4 toxicity; it consisted of Grade 3 or 4 hematologic toxicity and Grade 3 or 4 gastrointestinal toxicity. Toxicity outcomes were grouped into three: (1) any Grade 3 or 4 toxicity; (2) any Grade 3 or 4 hematologic toxicity; (3) any Grade 3 or 4 gastrointestinal toxicity.

### 4.3. SNP Selecting, DNA Extraction and Genotyping

We selected 28 SNPs of the WISP1 gene from the HapMap database according to the following criteria: (1) the minor allele frequency (MAF) of the SNP was >5% in the Chinese population; (2) haplotype tagger SNPs were selected by Haploview version 4.2 (Cambridge, MA, USA) using the pair-wise tagging with default settings (pair-wise *r*^2^ threshold = 0.8); and (3) SNPs in the promoter region, exon region and 3’UTR. Additionally, 2 SNPs that reported to be associated with cancer risk or clinical outcome were selected.

Genomic DNA of all subjects were isolated from the peripheral blood sample using the FlexiGene DNA Kit (Qiagen, Hilden, Germany) and stored at 4 °C until use. Genotyping was conducted by Sequenom’s MassARRAY system (Sequenom, San Diego, CA, USA).

### 4.4. Statistical Analysis

Toxicity outcomes were dichotomized by the presence or absence of Grade 3 or 4 toxicity. χ^2^ test and Student’s *t*-test were used to determine the differences in sex, age, smoking status, histology, stage and ECOG between these two groups. Unconditional logistic regression was performed to estimate the association of toxicity outcome with WISP1 polymorphisms by calculating odds ratios (OR) and their 95% confidence intervals (CI) adjusted by the covariates. The *p*-value was two-sided, and *p* < 0.05 was considered statistically significant. All association analyses were conducted by three models. The additive model is for the additive effects of SNPs, and the direction of the regression coefficient represents the effect of each extra minor allele. Dominant and recessive models are tests for the minor allele with two of the classes pooled. That is, if A is a minor allele and a is the major allele, the dominant model means (AA, Aa) *versus* aa, and the recessive model means AA *versus* (Aa, aa). The aforementioned statistical analyses were performed using PLINK [36] and SPSS 18.0 (SPSS Inc., Chicago, Illinois, USA).

## 5. Conclusions

We considered that genotypes of *WISP1* may be used to predict the platinum-based chemotherapy toxicity in lung cancer patients.

## Supplementary Materials

Supplementary materials can be found at: http://www.mdpi.com/1422-0067/15/11/21011/s1.
